# Experimental Framework for the Setup and Validation of Individualized Bone Conduction Hearing Computational Models

**DOI:** 10.3390/biomimetics10110738

**Published:** 2025-11-04

**Authors:** Johannes Niermann, Ivo Dobrev, Linus Taenzer, Christof Röösli, Bart Van Damme, Flurin Pfiffner

**Affiliations:** 1 Department of Otorhinolaryngology, Head and Neck Surgery, University Hospital Zürich, University of Zürich, 8091 Zürich, Switzerland; 2 Laboratory of Acoustics/Noise Control, Empa, 8600 Dübendorf, Switzerland

**Keywords:** bone conduction hearing, experimental validation, subject-specific models, computed tomography, laser Doppler vibrometry

## Abstract

In bone conduction (BC) hearing, sound is transmitted directly to the cochlea via skull vibrations, bypassing the outer and middle ear. This provides a therapeutic option for patients with conductive or mixed hearing loss and single-sided deafness. Although finite-element models have advanced understanding of the mechanisms underlying BC, progress toward personalized treatment strategies remains limited by a lack of standardized, experimentally validated, subject-specific models. This study proposes a hierarchical validation framework to support the development and validation of individualized computational models of the human head under BC stimulation. The framework spans four anatomical levels: system, subsystems, structures, and tissues. This approach enables systematic acquisition of data from intact cadaver heads down to isolated material domains. To demonstrate the applications of the framework, an experimental study was conducted on a single cadaver head, targeting three levels: the intact head (system), extracted bone pieces (structures), and isolated cortical layers (tissues). Subsystems were not addressed. High-resolution photon-counting computed tomography (CT) and energy-integrating cone-beam CT were used to acquire anatomical data. One-dimensional laser Doppler vibrometry was used to capture vibrational responses of bone pieces and cortical layers under wet and dry conditions. Representative results were analyzed to assess the impact of preparation state on resonance behavior. Comparative analysis showed that photon-counting CT provided superior structural resolution compared with energy-integrating cone-beam CT, particularly at the full-head (system) level. Vibrational measurements at the structure and tissue levels from the same anatomical region revealed broadly consistent resonance vibration patterns, enabling comparison of resonance frequencies. The influence of hydration state and thickness reduction on vibrational behavior was highlighted. The proposed framework provides a scalable methodology for validation of subject-specific BC models with the potential for more accurate BC simulations based on the hypothesis of functional variability rooted in anatomical variability. Obvious use cases would include the development of improved hearing aid designs and personalized treatments. In parallel, a successful correlation of anatomical and functional variability can serve as inspiration for design principles of metamaterials.

## 1. Introduction

Bone conduction (BC) hearing refers to the transmission of sound to the cochlea via skull vibrations. From a clinical perspective, BC hearing aids (BCHAs) are indicated for the treatment of conductive and mixed hearing loss, as well as single-sided deafness [1], as BC bypasses the outer and middle ear. Various technological methods exist for the design of BCHAs, but in all cases, vibrations travel from the mechanical excitation point to the inner ear, where they are converted into neural signals by the organ of Corti within the cochlea [2].

BC hearing sensation is thought to result from distinct mechanical transmission pathways [3]; the most important being the inertia of the cochlear fluid. The mechanisms underlying BC transmission of sound to the inner ear are complex; thus, quantification of the contributions of BC to auditory perception remains an active field of research [4]. A more comprehensive understanding of these mechanisms could lead to the development of personalized BC treatment strategies with enhanced BCHA designs.

Since the mid-2000s, finite-element (FE) modeling has been an essential tool for the investigation of the complex biomechanics of BC in the human head. Early models sought to capture the basic mechanical behavior of the skull under BC stimulation but lacked anatomical detail and validation. A notable early example is that of Taschke & Hudde (2006) [5], who developed a head model from computed tomography (CT) scans at 1 mm resolution. A non-linear grayscale to Young’s modulus mapping was employed to assign spatially heterogeneous stiffness to the bone domain. However, the model was limited to bone and skin material domains and was not validated experimentally. Other approaches have focused on isolated subsystems of the auditory pathway. For instance, Böhnke and Arnold (2006) [6] used their previously developed micro-CT-based FE cochlear model [7] to simulate the response to harmonic pressure. Similarly, Brummund et al. (2014) [8] modeled the outer ear based on the Visible Human Project^®^ database of the U.S. National Library of Medicine, using cryosectional images of a female adult with 0.33 mm voxel resolution to investigate the occlusion effect. These models offered valuable insight into structures closer to hearing sensation but lacked holistic interactions between BC transmission paths.

To overcome the limitations of previous approaches, researchers developed the anatomically detailed “LiUHead” whole-head FE model. The geometry of this model was also based on the Visible Human Project^®^. The initial 2014 version replicated an experimental setup of a polyurethane-filled dry human skull featuring a homogeneous bone domain and was validated using data from the literature on mechanical point impedance at excitation and cochlear promontory acceleration [9]. In 2016, the model was extended to include eight distinct material domains, comprising cortical and diploë bone layers, soft tissue, cartilage, cerebrospinal fluid, brain, eyes, and simplified inner ears [10]. This updated model matched trends in experimental BC data from previous human cadaver head studies. Further developments integrated a detailed cochlear submodel from micro-CT images of a human temporal bone [11].

The FE models described above have been key to our understanding of BC mechanisms, including the dominant role of bone in power transmission to the cochlea, particularly the dense cortical bone [12], and have been used in comparisons of BC devices under controlled conditions [13]. The FE models have also enabled the replication of cadaver head experiments. Specifically, the cochlear submodel enabled direct comparison of simulated intracochlear pressures against cadaver data and even exploration of the motion of otic capsule walls based on computational data [4].

However, despite advances in computational BC models, experimental validation remains scarce or non-existent. Classic validation has been based on data from the literature that is directly relevant to hearing sensation or excitation [8,9,10]; however, the material domains used in studies to date have been strongly homogenized, and limited applicable validated material parameters have been available.

The macroscopic mechanical properties of bone are based on its hierarchical structure [14]. Numerous studies have been conducted to characterize the mechanical properties of bone, yet, in particular, frequency-dependent damping still remains largely unexplored in the audible frequency range [15].

Recently, efforts have been made to identify elastic material properties of cranial cortical and diploë bone layers in the auditory range by fitting subject-specific simulations to experiments [16]. However, the identified properties were mostly frequency-independent and from only a single dried skull. Physiologically, the bone is saturated with water, which considerably affects its mechanical properties [17].

Substantial inter-subject differences in cortical bone and diploë thickness distributions have been reported in a study including ten fresh post mortem human surrogates [18]. The identified bone phase Young’s modulus of the same subjects showed no significant inter-subject differences [19]. However, significant spatial differences were found.

Another study showed that in an in vivo population of 30, the inter-subject functional variability in BC response was on average 20 dB in the frequency range from 0 to 5 kHz, with an average intra-subject variability of 2.7 dB [20]. Instantaneous velocity patterns of cadaver head skull surfaces under BC reconfirmed the observation of functional inter-sample variations [21]. Numerical investigations of BC response with varying stiffness of the cortical bone domain indicated functional dependence on anatomy rather than material properties [4]. Given the above-mentioned functional variability paired with the anatomical variability and limited relevance of material properties, individualized models present a promising approach for more accurate quantification of pathway contributions.

At the same time, these variabilities can be viewed as a source of inspiration for design principles of dynamic mechanical metamaterials and BCHA implants. Bio-inspired acoustic [22] and elastic metamaterials [23] have been shown to be effective tools to manipulate wave propagation through local resonance and Bragg scattering band gaps. Bone microstructure has been successfully used as inspiration for biomimetic stiffness-matched implants [24,25].

A correlation of locally resonant microscale trabecular porosity and mesoscale cortical and trabecular thickness, as well as hydration-dependent properties with frequency-dependent macroscopic resonances and attenuation, could lead to materials with applications in sensing and vibration control. Crucially, the same mapping could enable personalized BCHAs in which implant geometry and drive-axis orientation are tuned to maximize cochlear accelerance.

Although there is no standard for the development of validated individualized biomechanics models, methodological guidelines have been formulated by Henninger et al. (2010) [26], including examples of successful studies with clinical implications.

This study initiates the development of a comprehensive framework of experimental methods to support the systematic construction and validation of subject-specific computational models of the human head under BC stimulation. By employing a hierarchical validation strategy, experimental data across multiple anatomical levels are acquired, ranging from intact cadaver heads to isolated bone pieces and cortical tissue layers. This approach not only maximizes the information extracted from each specimen but will also enable incremental validation of model components under increasingly controlled conditions.

To demonstrate the applications of the proposed framework, imaging modalities are compared, and exemplary functional measurements across selected hierarchical levels for one cadaver head are presented. These could serve as foundational building blocks for subject-specific model development and validation. The examples presented here offer insights into model individualization possibilities and the influence of material condition and sample preparation on vibrational behavior at the structure and tissue levels.

## 2. Methodology

### 2.1. Hierarchical Validation Approach

**Figure 1:**
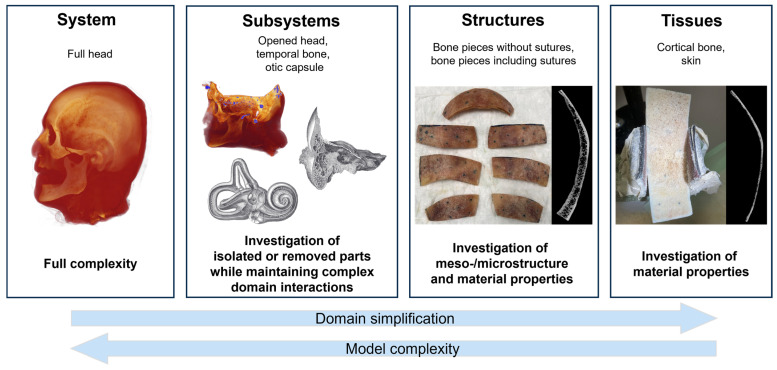
Hierarchical validation approach (following Henninger et al. (2010) [26], originally adapted from Oberkampf et al. (2004) [28]) for biomechanical problems. Illustrations of the temporal bone and cochlea subsystems were adapted from Gray & Carter (1918) [29].

Biological systems such as the human head involve numerous complex material domain interactions. To address this complexity in modeling, we refer to the modular hierarchical validation framework for biomechanical models proposed by Henninger et al. (2010) [26]. Figure 1 illustrates how the framework can be applied to human cadaver heads under BC. Cadaver heads remain a vital proxy for in vivo studies in BC research, as they enable measurement procedures that would be impossible in living subjects. Cadaver heads are considered to express comparable BC response as living humans [27].

The framework comprises four levels in a hierarchical sequence of data collection and model validation: system, subsystems, structures, and tissues. This hierarchical approach allows data acquisition from cadaver heads to be maximized, enabling subject-specific data correlation across multiple levels.

At the system level, a model that has been validated according to the proposed hierarchical framework allows investigation of the interactions of all represented subdomains (e.g., skull bone layers, skin, and cerebrospinal fluid). Thus, this phase represents the final step from a model validation perspective but the initial step from a data-gathering perspective.

Typical functional data gathered from full heads may include skull bone surface velocity motion [21,30,31,32], cochlear promontory [33] and stapes motion [34,35], intracranial pressure [36], and intracochlear sound pressure (ICSP) [34,37,38,39] under varying stimulation conditions [33] and mechanical point impedance at stimulation sites [40]. Subsequent data-gathering phases for the same sample focus on the lower levels in the hierarchy.

Subsystems such as the skull base and temporal bones contain much of the complexity of intact cadaver heads, but can be defined to simplify data gathering at points of interest. For example, in the case of the skull base, the skull is opened, and the brain is removed to allow optical access to the medial side of the bone surface of the skull base. The skull base subsystem remains representative of the vibrational behavior of the skull, particularly within the temporal bone and otic capsule [4]. This also provides access to bone surfaces within a few millimeters of the otic capsule, enabling the collection of three-dimensional (3D) velocity data. In addition, the cadaver heads can be modified to eliminate elements of BC pathways or material domains and to quantify their influence (e.g., the skin, brain, or cerebrospinal fluid). Specifically, the effects of the skull contents on skull bone motion have been experimentally described [41] and are available for model validations at a later stage.

At the structure level, bone pieces, for instance, can be used to investigate the relevance of subject-specific meso- and microstructure, such as cortical and trabecular bone and suture morphology, to the accuracy of model results. In addition, the bone pieces can be dried and rehumidified to enable exploration of the effects of water content on the effective damping within skull bone. In each condition of the bone pieces, functional data such as surface motion under BC stimulation can then be collected.

Tissues such as cortical bone or skin can be used to determine material properties of the model on an individual material domain level. In the case of the cortical bone, cortical tissue layers can be extracted from bone pieces and investigated under wet and dry conditions, similar to full-thickness bone pieces.

In parallel with functional information, individualized model geometries require subject-specific anatomical data, which would ideally be acquired consistently across all hierarchical levels. Such comprehensive validation is crucial for the prediction of responses in experimentally inaccessible domains, such as third-window motion or patient-specific in vivo responses. Furthermore, it provides a robust validation framework throughout model development. This approach means the development process can begin at lower levels of the hierarchy, enabling an efficient modeling pipeline to be established. However, it involves a complex experimental process with multiple temporal dependencies.

**Figure 2:**
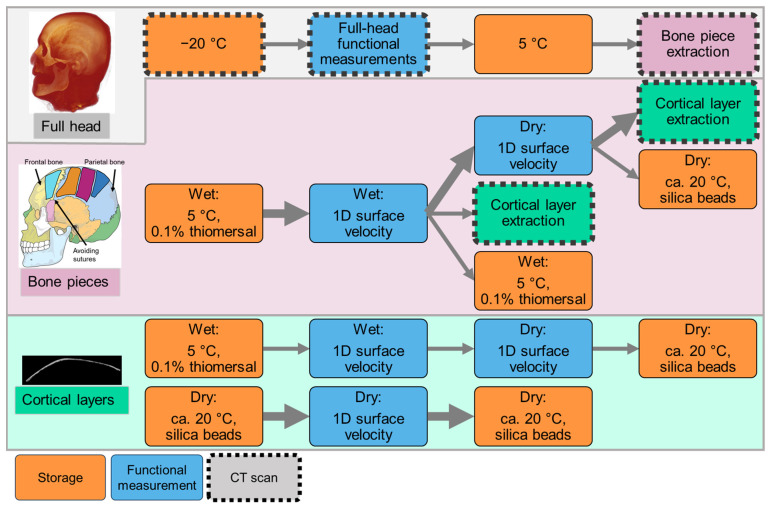
Measurement protocol for the cadaver head, detailing sample handling across hierarchical levels: full head (system), bone pieces (structures), and cortical layers (tissue). No subsystem was investigated in this case. Sample storage steps are marked in orange, and functional measurements are indicated in blue. Sample states documented via CT scanning are outlined with a dashed black contour. The timeline proceeds from top to bottom and from left to right as indicated by arrows. Big arrows correspond to the measurement protocol of the representative bone piece selected to present functional measurements. The skull illustration was adapted from Villarreal (2017) [43].

In this work, the framework is illustrated with an example procedure that applies only a subset of the possible measurements described above to a single specimen. The applied measurement protocol is presented in Section 2.3 and outlined in Figure 2. Section 2.4 outlines sample preparation steps corresponding to that example. The tools and methodologies used for data generation are described in Section 2.5, and Section 2.6 describes how the data were processed. It should be noted that procedures may differ across specimens. Selected comparisons of anatomical measurements at the full head and bone piece levels, as well as functional measurements at the bone piece and cortical layer levels, are presented in Section 3.

### 2.2. Cadaver Head Sample

The cadaver head of a 66-year-old female was obtained from Anatomy Gifts Registry (Hanover, MD, USA). The head was fresh-frozen and remained in a controlled −20 °C freezer until shortly before the start of the experimental procedure. Freezing and thawing have little effect on the mechanical properties of bone [42]. Energy-integrating cone-beam CT (EI-CBCT) was used to assess sample quality and ensure structural integrity and the absence of metallic artifacts. This study was approved by the Ethical Committee of Zürich (KEK-ZH–Nr. 2018–02,014).

### 2.3. Measurement Protocol

#### 2.3.1. Full Head

Although the head had been pre-scanned using EI-CBCT (NewTom VGi evo, CEFLA s.c., Imola, Italy), it was scanned again before the experiments in a frozen state using photon-counting CT (PCCT) to investigate image quality differences in the anatomy for model setup. The head was out of the freezer for as short a time as possible and was contained in a cooled box. After defrosting, all functional full head measurements were conducted within 1 week. Between measurements, the head was stored at 5 °C. BC stimulation was applied at multiple standard BCHA stimulation locations behind the ear; a Baha SuperPower 5 (Cochlear Ltd., Melbourne, Australia) was used for this purpose owing to its high output [44,45].

The measured functional data included ipsilateral cochlear promontory 3D point velocity [36], ICSP [34], and superior skull 3D surface velocity [21]. Previous studies have reported these data using the same procedures on multiple specimens; therefore, they are not presented in the current work. Nevertheless, the subject-specific validation framework will incorporate them when model results are available. To enable registration of velocity and CT data, the positions of previously applied metal reference markers were documented both in the velocity measurement coordinate system and in EI-CBCT data, as in previous work [46]. In addition, these EI-CBCT scans were used to document excitation location positions and structural changes due to sample preparation procedures, such as drilling holes.

#### 2.3.2. Bone Pieces

**Figure 3:**
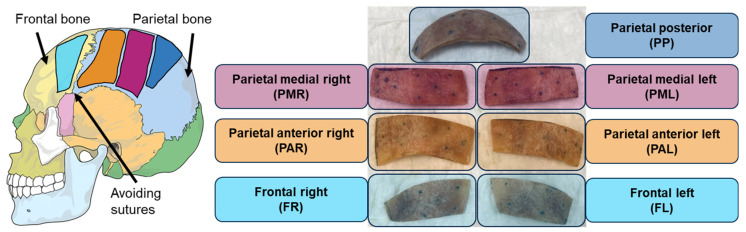
Approximate positions of extracted bone pieces (structures) named after the anatomical regions in the full head. The skull illustration was adapted from Villarreal (2017) [43].

After the full head measurements, bone pieces were extracted from previously defined positions as illustrated in Figure 3. The bone pieces were cut from the superior skull in the same area in which the full skull 3D surface velocity measurements were performed. Owing to immediate availability, the extracted bone pieces were scanned using EI-CBCT for structure assessment and future model generation. Previous investigations have been conducted on an unrelated bone piece; the results indicated that at this sample size, EI-CBCT scans offer resolution comparable with that of PCCT. After extraction, the bone pieces were stored in a 0.1% thiomersal solution in a fridge controlled at 5 °C to prevent them from being degraded. Certain wet bone specimens underwent a drying process to remove moisture and thereby reduce effective damping. Chemically dried specimens were stored in a sealed container with silica beads to facilitate desiccant-based drying. Functional bone piece measurements were performed to quantify one-dimensional (1D) surface velocities under wet and dry conditions using different BC excitation methods.

**Figure 4:**
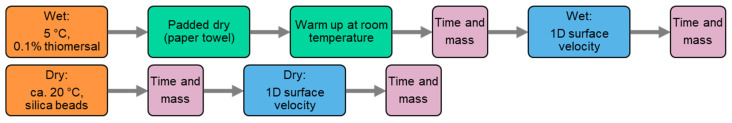
Measurement protocols for bone piece (structure) and cortical layer (tissue) measurements under wet and dry conditions.

Qualitatively, all bone pieces exhibited vibration responses similar to those presented in a previous study [16]. The parietal medial right (PMR) bone piece (as defined in Figure 3) was selected as a representative example to present functional measurements under both wet and dry conditions. BC stimulation was applied using a piezo actuator (PA) (PC4WL, Thorlabs Inc., Newton, NJ, USA), as it is considered easier to model for small sample sizes than a BCHA due to its simpler coupling and lower mass, while maintaining high signal levels across a wide bandwidth. The PA was mounted at a corner of the peripheral surface, with its position kept as consistent as possible across conditions. During functional measurements, the specimens were placed on foam to approximate a free-fixation boundary condition. The specific procedure for functional measurements under both wet and dry conditions is outlined in Figure 4.

The wet bone piece was taken out of the 5 °C thiomersal solution, patted dry with a paper towel to remove excess water, and then rested at room temperature for about 30 min to reduce any temperature-dependent effects. Mass and time were measured before and after the 1D surface velocity measurement. Similarly, for the dry piece, mass and time were measured before and after the functional measurement.

#### 2.3.3. Cortical Layers

According to the overall protocol presented in Figure 2, PMR and parietal medial left (PML) samples (as defined in Figure 3) were selected based on the presence of clearly distinguishable layers in the EI-CBCT images and ground down to their outer cortical layers. The geometries of the resulting cortical layers were documented using EI-CBCT. At the time of extraction, the PML specimen was wet, whereas the PMR specimen had already been dried. The PML cortical layer was investigated under both wet and dry conditions. Functional measurements were conducted on the cortical layers to quantify 1D surface velocities under wet and dry conditions using different BC excitation methods.

The dry PMR cortical layer is presented as a representative example of the functional measurement results. The experimental setup was identical to that used for the corresponding PMR bone piece measurements described in Section 2.3.2, with a PA used to induce BC at a corner of the peripheral surface and the specimen placed on foam. The measurement procedure followed the dry condition protocol outlined in Figure 4.

### 2.4. Sample Preparation

#### 2.4.1. Full Head

The cadaver head was defrosted at 5 °C for approximately 60 h before experimentation. Whole-head preparation involved several surgical and procedural steps, including mastoidectomy to expose the cochlear promontory, cochleostomy for ICSP sensor access to the scalae of the cochlea, and skin removal to expose bone surfaces [46]. Fiducial markers were attached with cyanoacrylate adhesive (Sekundenkleber Flüssig, Uhu Holding GmbH, Bühl, Germany) into pre-drilled 1 mm holes in the superior cranium. Retroreflective beads were applied using lacquer spray to enhance laser reflectivity for velocity measurements. Additional preparation steps included drilling for application of the BC stimulation (Baha^®^ Connect System, Cochlear Ltd., Melbourne, Australia), insertion of pressure sensors into the cochlea, installation of a hydration pump, and configuration of the fixation setup for measurements, as defined by Dobrev et al. (2023) [46].

#### 2.4.2. Bone Pieces

Each specimen measured approximately 10 cm by 3 cm and contained both cortical and diploë layers but no sutures. These dimensions were selected to ensure a sufficient number and variety of mode shapes under BC excitation to serve as features for modeling and validation. As the specimen surfaces had been measured previously in full-head experiments, retroreflective beads were removed using Dremel polishing tools with 80–300 grit brushes to minimize CT imaging artifacts. For vibration experiments, new beads were reapplied using lacquer spray.

Specimens designated for drying were chemically treated in a 20% hydrogen peroxide solution for approximately 12 h. The PA was mounted on the wet PMR specimen using cyanoacrylate adhesive (Sekundenkleber Flüssig, Uhu Holding GmbH, Bühl, Germany) and on the dry PMR specimen using phenyl salicylate, which solidifies rapidly without the introduction of moisture and provides a high degree of stiffness. After measurements, the PA was detached from the specimen.

#### 2.4.3. Cortical Layers

For tissue-level investigations, selected bone specimens were secured laterally in a vice and further ground down to isolate single cortical layers using an oscillatory grinding tool (AIZ 32 RT5 Carbide, Robert Bosch GmbH, Stuttgart, Germany). As shown in Figure 1, the vice was fitted with rubber pads covered in 120-grit sandpaper to provide adequate clamping force while minimizing structural damage. As the specimen surfaces had been previously measured in bone piece experiments, retroreflective beads were removed using Dremel polishing tools with 80–300 grit brushes to prevent CT imaging artifacts. For vibration experiments, new beads were reapplied using lacquer spray.

The PMR cortical layer designated for drying was chemically treated using the same procedure as that used for the bone specimens. The PA was attached to the dry PMR cortical layer using the same method as that used for the dry bone specimen, employing phenyl salicylate. Following measurement, the PA was detached from the specimen.

### 2.5. Measurement Methods

#### 2.5.1. Structure

Owing to the dominant role of bone in BC and the feasibility of frozen-state imaging, subject-specific anatomical data were acquired via CT. EI-CBCT scans were performed according to standard clinical head CT protocols (NewTom Vgi evo). The PCCT scans were performed using a dedicated ex vivo bone-optimized protocol on a PCCT scanner (NAEOTOM Alpha, Siemens AG, Berlin, Germany). Fields of view (FOVs) were minimized to sample dimensions, and the resulting voxel sizes were determined.

#### 2.5.2. Pressure

Intracochlear sound pressure (ICSP) measurements quantify the transmission of bone-conducted stimulation to the inner ear and have been recorded using custom-made pressure sensors inserted into both the scala vestibuli and the scala tympani, as described in previous work [4,21,36].

#### 2.5.3. Velocity

Laser Doppler vibrometry was used to obtain non-contact velocity measurements; this method avoids any alteration of the natural structural response, while providing the sensitivity required to resolve the minute velocities associated with bone conduction stimulation. The three components of the velocity on the cochlear promontory and the 3D surface velocities of the superior cranium in the intact head were measured using a 3D laser Doppler vibrometer (CLV-3000, CLV-3D, Polytec GmbH, Waldbronn, Germany), as in previous work [4,21,36]. Functional 1D surface velocity measurements on bone pieces and cortical layers were conducted using an SWIR scanning laser Doppler vibrometer (SLDV, Scan-Sense, OptoMET GmbH, Darmstadt, Germany).

For the representative PMR specimen (Figure 3), the excitation was applied using a chirp signal from 1 Hz to 20 kHz, with responses recorded at a spectral resolution of 12,800 lines and averaged over four measurements per point. Amplification was held constant across all conditions. On the peripheral surface, the bone pieces were measured at 334 points; for the cortical layer, the number of points was reduced to 240 owing to constraints on measurement duration and data volume. Measurement grids overlaid on images of the specimens with the attached PA are provided in Appendix A.

The pre-amplification driving voltage and velocity time signals were recorded using the internal data acquisition system of the SLDV. For each measurement point, the SLDV software was used to compute complex-valued frequency response functions (FRFs) with the pre-amplification driving voltage as input and the velocity as output, as well as frequency-dependent coherence values for further processing. The coordinates of all measurement points were also recorded in the SLDV coordinate system.

#### 2.5.4. Mass

As mass variation is a fundamental parameter governing motion, its documentation is essential for reliable assessment of BC phenomena, particularly in controlled scenarios such as water content removal or cortical layer extraction. The mass of bone pieces and cortical layers was measured using an analytical balance (type SE 422, VWR, Radnor, PA, USA). Each reported mass corresponded to an average of at least three measurements, with the specimen repositioned between measurements.

### 2.6. Data Processing

#### 2.6.1. Image Processing

For image modality comparisons, datasets were processed in Amira 6.5 (Thermo Fisher Scientific, Waltham, MA, USA). Grayscale values in the CT datasets were thresholded between 200 and 2500 HU to enhance cortical and trabecular bone visibility for subsequent analysis. Volumetric alignment was then performed: full cadaver head scans were manually aligned owing to their high spatial complexity, whereas cranial bone piece scans were subjected to automated 3D registration to ensure accurate anatomical matching. Comparative analysis was based on anatomically corresponding cross-sectional slices from both modalities. Owing to differences in slice counts and thicknesses, slices were manually selected in Amira to represent the closest anatomical regions, without necessarily having an exact 2D correspondence of shapes.

#### 2.6.2. Functional Data Processing

For the SLDV data, complex FRFs were pre-processed using a three-stage filtering procedure; this was performed across all points at each frequency, resulting in a spatial masking that was unique at each frequency. The stages included global coherence filtering, which excluded points with insufficient coherence; global amplitude filtering, which removed extreme magnitude outliers using symmetric percentile thresholds; and local consistency filtering, which rejected points (at individual frequencies) exhibiting excessive discrepancy relative to neighboring points. The resulting spatial masks were combined multiplicatively. Owing to the lower signal-to-noise ratio at the low-frequency cortical layer resonances, more inclusive filtering settings were applied relative to the bone pieces to ensure retention of more low-frequency data. Specifically, thresholds for global coherence and local consistency filtering were lowered for cortical layer samples, with global amplitude filtering remaining constant.

An initial overview was obtained by calculating point-averaged magnitudes from the pre-processed complex-valued FRFs across the measured frequency range. Resonance peaks in the averaged FRF curves were automatically identified using a peak detection algorithm (findpeaks in MATLAB R2024a) with a constant parameter set across conditions. Nine operational deflection shapes (ODSs) in the form of phase maps were processed for each condition. For the wet and dry bone piece conditions, the frequencies of the first nine detected peaks were used. For the cortical layer condition, the first three ODSs were manually selected to match the corresponding bone piece patterns owing to low-frequency noise; the remaining six corresponded to the first six detected peaks. Global phase shifts were manually adjusted per plot to improve the visualization of correspondence between sample conditions.

## 3. Results

### 3.1. Imaging Modalities for Model Setup

#### 3.1.1. Full Head Visualization

**Figure 5:**
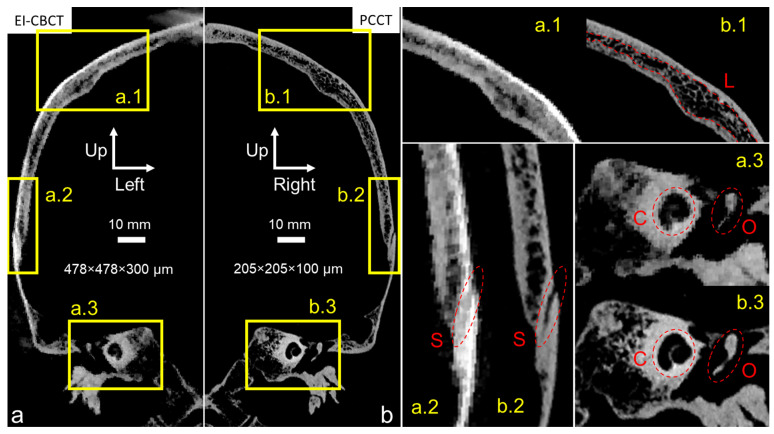
Comparative visualization of mirrored coronal plane reconstructions of an intact frozen cadaver head acquired for subject-specific model construction using (**a**) EI-CBCT (voxel size 478 × 478 × 300 µm) and (**b**) PCCT (voxel size 205 × 205 × 100 µm). Brightness and contrast in image a were adjusted to approximate those in image (**b**). Magnified regions highlight corresponding anatomical structures: (**a.1**,**b.1**) depict the parietal bone, including characteristic cortical bone and diploë layers (L); (**a.2**,**b.2**) show the transitional suture from parietal to temporal bone (S); and (**a.3**,**b.3**) show the otic capsule (C) and a middle ear ossicle (O).

The PCCT scan of the cadaver head (Figure 5b) exhibited markedly higher spatial resolution compared with the EI-CBCT scan (Figure 5a), as evidenced by sharper cortical outlines and finer trabecular detail. In the PCCT reconstruction, fine trabeculae (wall thickness < 350 µm) within the skull plates were clearly visualized (Figure 5(b.1)), and transitions from cortical to diploë layers were distinct in the superior cranium (Figure 5(b.1) (L)). Sutures such as the squamous suture between the parietal and temporal bones (Figure 5(b.2) (S)) and bony structures of the ear, including the cochlear portion of the otic capsule (Figure 5(b.3) (C)) and the middle ear ossicles (Figure 5(b.3) (O)), were also well delineated.

At a comparable FOV, the EI-CBCT scan had an approximately 2.5-fold larger voxel size than the PCCT scan, resulting in visibly reduced resolution. Trabecular structures (Figure 5(a.1)) and the parietotemporal suture (Figure 5(a.2) (S)) appeared blurred, preventing discrimination of individual trabeculae and limiting precise suture segmentation. Cortical-to-trabecular transitions were less well defined, complicating the distinction between cortical and diploë layers. Although the cochlea (Figure 5(a.3) (C)) and ossicular outlines (Figure 5(a.3) (O)) remained visible, their boundaries were ambiguous. In addition, imaging artifacts were present near the cranial vertex (Figure 5a).

#### 3.1.2. Bone Piece Visualization

**Figure 6:**
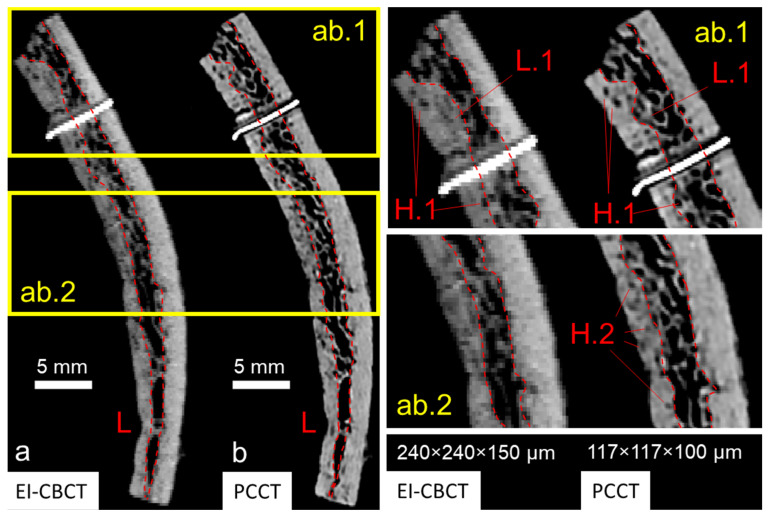
Comparative visualization of cross-sectional reconstructions of an extracted superior cranial bone piece acquired using (**a**) EI-CBCT (voxel size 240 × 240 × 150 µm) and (**b**) PCCT (voxel size 117 × 117 × 100 µm) displayed at anatomically corresponding slice locations. The cross-section shows characteristic layered cortical bone and diploë distributions (L) and a metal reference marker. Magnified regions (**ab.1**,**ab.2**) highlight differences in subjectively defined transitions from cortical bone to diploë (L.1) and visibility of larger (H.1) and smaller (H.2) pores within cortical bone.

Overall, for a comparable FOV, the PCCT scan demonstrated superior spatial resolution (approximately two-fold greater based on voxel size) relative to the EI-CBCT scan. Although small trabecular elements (wall thickness < 300 µm in EI-CBCT, <450 µm in PCCT) were identifiable in both datasets, trabecular outlines in the PCCT image (Figure 6(ab.1,ab.2)) were sharply defined, whereas those in the EI-CBCT image (Figure 6(ab.1,ab.2)) appeared diffuse. Larger cavities within the bone matrix were visible in both modalities (Figure 6(ab.1) (H.1)), but smaller cavities were clearly resolved in the PCCT image, whereas they were indistinct in or absent from the EI-CBCT image (Figure 6(ab.2) (H.2)). In the PCCT scan, a well-defined bilateral gap between the metal marker and bone tissue was apparent, whereas in the EI-CBCT scan the gap appeared only as a faint, low-density line, and the marker was considerably larger.

The lower resolution and blurring in EI-CBCT led to overestimation of high-density areas compared with PCCT, resulting in an increase in cortical layer thickness of the order of 50% in extreme regions based on preliminary analysis (Figure 6(ab.1) (L.1)). Although both modalities enabled cortical–trabecular differentiation, the delineation of cortical and diploë layers was inconsistent between EI-CBCT and PCCT.

### 3.2. Functional Data for Bone Piece and Cortical Layer Level

**Figure 7:**
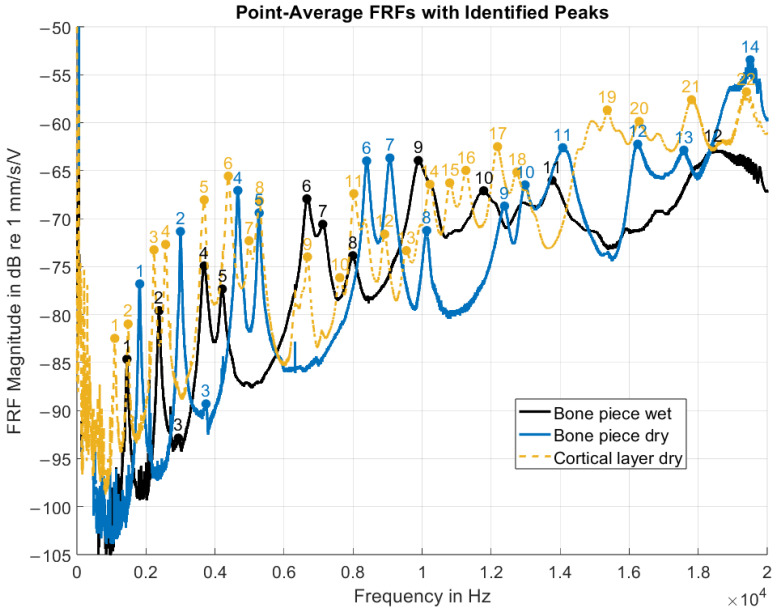
Point-average FRF magnitudes of the PMR sample under the three experimental conditions. The FRFs were defined with the driving voltage (pre-amplification) as input and the velocity response as output. Prominent spectral peaks were automatically identified and are sequentially numbered in order of increasing frequency.

The point-average FRF magnitudes (Figure 7) showed an overall increase with frequency, punctuated by multiple distinct resonance peaks. As these curves represented spatial averages over all measurement points, the peaks corresponded to frequencies at which a substantial portion of points exhibited elevated velocity responses relative to neighboring frequencies, indicating global structural resonances. Peak detection identified 12 resonance peaks for the wet bone piece, 14 for the dry bone piece, and 22 for the dry cortical layer. Several visually apparent peaks were not detected by the algorithm, particularly in the cortical layer dataset. Data quality was reduced at both low frequencies (0–200 Hz) and at the upper end of the spectrum (18–20 kHz), limiting robust automated peak identification in these ranges. Factors that are likely to have contributed to this reduction in data quality include low actuator signal-to-noise ratio, low-frequency setup noise, and boundary condition effects that may have obscured the true material response.

Under the wet and dry full-thickness bone conditions, a similar distribution of resonance peaks was obtained, especially in the 1–11 kHz range. The spectral pattern for the wet condition seemed to be preserved under the dry condition, but was shifted toward higher frequencies. However, confirmation of the correspondence between individual resonances required complementary spatial analysis of vibration patterns.

**Figure 8:**
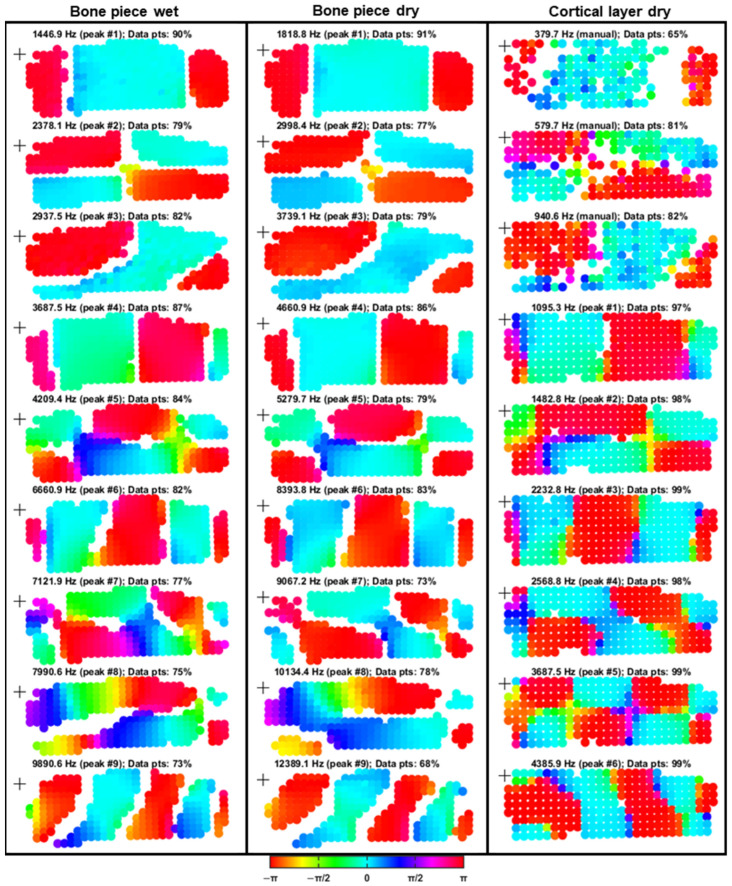
FRF phase maps of the PMR samples under the outlined experimental conditions. The FRFs were defined with the driving voltage (pre-amplification) as input and the velocity response as output. Each column contains nine phase maps corresponding to selected resonance frequencies, with measurement points color-coded according to phase based on the HSV colormap. Peak numbers correspond to those identified in Figure 7. For the cortical layer condition, the first three resonances were manually selected to be visually aligned with the vibration patterns of the first three resonance peaks observed in the full-thickness bone piece conditions. The percentages of remaining data points after filtering are expressed in the title of each plot. The excitation locations are indicated by black crosses in each plot.

The FRF phase maps (Figure 8) showed broadly consistent vibration behavior across all three experimental conditions, with spatial complexity generally increasing at higher resonance frequencies. For the wet and dry bone piece conditions, all ODSs agreed well, with slight inconsistencies observed for ODSs 5, 7, and 8 owing to phase gradients and less distinct nodal lines.

Despite the geometrical difference in thickness between the bone pieces and the cortical layer, a comparative analysis was conducted to assess the influence of sample preparation on vibrational measurements. For the cortical layer, a lighter filtering approach was applied to preserve more low-frequency information. However, despite these adjustments, resonance patterns at manually selected frequencies within the noisy 0–1 kHz range remained less distinct than those of the bone pieces. At 380 Hz, 65% of the original data points were retained when the adjusted filtering settings were used. By comparison, application of the same filtering parameters as those used for bone piece conditions would have resulted in only 27% of data points remaining at this frequency. Nonetheless, clear phase distributions enabled resonance correlation: ODSs 1–7 of the cortical layer aligned well with the corresponding ODSs from the bone piece conditions, with minor inconsistencies again noted for ODSs 5 and 7. The vibration pattern of ODS 8 was not directly comparable, and ODS 9 exhibited a similar (but mirrored) vibration pattern to that of bone piece ODS 9.

Quantitatively, the mean relative increase in resonance frequency from the wet to the dry bone piece condition was 0.337 octaves (26.27%), with a standard deviation of 0.011 octaves (0.74%). Although the ODSs of the cortical layer did not exhibit perfect alignment in all cases, the frequency shift was calculated to maintain methodological consistency. The transition from the dry bone piece to the dry cortical layer condition produced a mean decrease of −1.885 octaves (−72.92%), with a standard deviation of 0.082 octaves (5.83%).

Mass measurements also revealed substantial differences across conditions. The wet bone piece exhibited a decrease in mass during functional measurements (−1.36%), whereas both the dry bone piece (+0.09%) and the cortical layer (+0.12%) showed slight increases (Table 1). Based on initial mass values, the transition from wet to dry bone resulted in a −22.31% reduction in mass, whereas transitioning from the dry bone piece to the dry cortical layer produced a −64.45% reduction.

In summary, functional BC data revealed broadly similar vibration patterns between wet and dry full-thickness bone, with a frequency shift toward higher resonances observed under the dry condition. The cortical layer showed a greater number of identifiable peaks, particularly at higher frequencies, although some low-frequency resonances were less distinct owing to noise. Operational deflection shape comparisons confirmed the preservation of many resonance patterns across conditions, indicating that key global vibration modes persisted despite changes in hydration state or substantial bone mass removal from the specimen. However, at higher frequencies, peak detection identified non-correlating ODSs for the cortical layer.

## 4. Discussion

In the absence of established standards for experimentally validated individualized BC models, this work introduces a modular approach based on hierarchical validation. The results are expected to evolve iteratively through continued experimental data acquisition and model refinement, highlighting the scalability of the framework. Preliminary analysis of experimental results is presented as guidance for future steps.

### 4.1. Imaging Modalities and Model Geometry

Owing to the inverse nature of data acquisition versus model development, selection of an appropriate imaging modality is critical. EI-CBCT lacks sufficient resolution to reliably differentiate between cortical and diploic layers in full-head FOV imaging. This limitation also impedes the reliable extraction of other subject-specific anatomical features, such as the cochleae, which are essential for ICSP simulation. Consequently, PCCT is required at this scale. However, the limited availability of PCCT systems restricts its frequent use. EI-CBCT thus remains essential for documenting marker positions and anatomical alterations (e.g., post-mastoidectomy). Therefore, accurate volumetric registration between these imaging modalities is required to integrate anatomical modifications observed via one or several EI-CBCT scans into the model geometry obtained via a single high-resolution PCCT scan.

For isolated bone specimens (<10 cm in size), EI-CBCT may offer sufficient resolution for the extraction of subject-specific geometries of the cortical and diploic layers. However, its lower resolution relative to PCCT leads to discrepancies in layer thickness (cf. Figure 6(ab.1), L.1). Layer segmentations will likely introduce localized homogenization errors that could affect the agreement between experimental results and numerical simulations.

Kohtanen et al. (2021) [16] accurately matched modal frequencies with a mean error below 1.3% by defining cortical and diploë material domain boundaries based on HU thresholding applied across cross-sectional increments with voxel sizes of approximately 50 µm from micro-CT. By contrast, Alexander et al. (2019) [47] conducted an even higher-resolution structural analysis at voxel sizes below 10 µm, enabling the use of a statistical, porosity-based segmentation approach in which layer boundaries were determined by a pre-defined cutoff percentage. Nevertheless, the inherent biological variability of this system renders the problem fundamentally ill-posed, as subject-specific anatomical variations such as venous channels, cortical bone proliferations, and perforations (particularly within the inner diploic layer) can locally obscure or contradict the definition of three distinct layers.

To date, no systematic study has compared EI-CBCT and PCCT in this context. Therefore, a detailed investigation of bone specimens combined with simulation-based validation represents a logical next step. A potential approach involves generating multiple segmentations of the same specimen using both imaging modalities and a range of segmentation strategies. In addition, micro-CT [16,18,47] could be employed to generate a high-resolution baseline model that could serve as a reference (ground truth) for evaluation of segmentation accuracy and structural fidelity. Subsequent modal analyses, either assuming constant material properties or applying a material identification scheme similar to that used by Kohtanen et al. (2021) [16], could then be performed. The former approach is likely to be more practical, given the computational demands and stricter mathematical requirements for convergence and excitation definition associated with the latter.

### 4.2. Comparison of Functional Results

The comparison of velocity-per-voltage FRF metrics provides a preliminary assessment of the overall experimental procedure, in particular, the effects of the drying process and cortical layer extraction. The positive slope observed for the point-averaged FRFs can be attributed to the use of a constant driving voltage in conjunction with the decreasing (capacitor-like) electrical resistance of the PA excitation mechanism at higher frequencies. Notably, despite substantial alterations in sample condition resulting from drying and removal of the cortical layer, the ODSs remained largely consistent across all experimental states. Certain modes seemed to be dominated by specific deformation mechanisms, for example, bending in ODSs 1, 4, and 6, and torsion in ODS 2. However, given the geometric complexity of the bone specimens, the observed deformation patterns are likely to represent a superposition of multiple deformation modes.

The similarity in ODSs between the wet and dry bone conditions and the low standard deviation in global resonance frequency shifts suggest a relatively uniform reduction in water content throughout the structure. By contrast, the more pronounced differences in ODSs between the dry bone and dry cortical layer conditions, coupled with the increased standard deviation of resonance frequency shifts, highlight difficulties in making comparisons across these geometrically differing conditions. The observed differences may stem from variations in thickness, microcracks introduced during the process of cortical layer extraction, or limitations in the peak detection methodology.

Regarding the validation approach, it is important to recognize that vibration patterns are not expected to remain invariant across hierarchical levels. Variations in ODSs due to geometrical differences do not compromise the determination of material properties, as these can still be transferred to other hierarchical levels.

As FRFs capture the dynamic behavior of the entire system, including boundary conditions, the resonance frequencies observed in the bone piece conditions are likely to more accurately reflect the intrinsic structural resonances of the sample than those observed in the isolated cortical layer. This is primarily because of the higher mass ratio of the PA relative to the sample. The addition of a localized mass can alter the vibration patterns of a structure [48], and such effects must be accounted for when inferring material properties through comparison with numerical models. Furthermore, matching of the absolute vibration amplitudes between experiment and simulation would require a voltage-to-force calibration of the BC excitation source.

In the present study, ODSs were compared qualitatively through visual inspection; this provided a reasonable approximation for evaluating global vibration behavior. However, more rigorous quantitative comparisons of experimental and simulated vibration patterns will require implementation of appropriate registration and spatial interpolation procedures [46].

### 4.3. Individualization

Individualized models span a spectrum from anatomical fitting to microstructurally informed material assignment and thus require definitions that are aligned with the intended use of the model. Although all biomechanical models derived from CT-based geometry extraction may be subject-specific, their construction can vary substantially. For example, anatomical domain distributions and material properties of full-bone-thickness homogeneous models [9] differ markedly from multi-layer [10] material domain representations [16]. Accurate structural representation is especially critical in anatomical regions in which geometry (rather than material properties) primarily determines effective stiffness, as has been hypothesized for the base of the skull [4].

When considering individualized material properties, it is crucial to acknowledge the limitations of parameter identification based on fitting simulations to experimental data. Conversely, treating experimental results as absolute ground truth can lead to pitfalls. As demonstrated in Section 3.2, multiple factors, including intra-experimental variations (Table 1) and inter-experimental mass differences (e.g., differences due to varying drying states), can influence the mechanical response. In addition, microstructural damage introduced during sample preparation (e.g., by cutting, grinding, or drying) may reduce the effective stiffness of the specimen, thereby introducing systematic errors into the material identification process. As a result, the applicability of the identified properties is context-dependent and valid only within a limited range.

Similarly, system-level functional validation metrics, such as cochlear promontory motion or ICSP, are also confined to a specific range of validation accuracy. The strength of individualized models is their capacity to reduce variability in comparison with population-averaged models. Notably, increasing the assessed population size within an individualized modeling scheme can enhance the robustness and generalizability of conclusions drawn from the models.

## 5. Conclusions and Outlook

A logical next step in the development of individualized BC models will be the generation of preliminary simulation results, starting with samples lower on the structural hierarchy, namely, cortical layers and bone pieces. Once a robust model validation pipeline is in place, model result differences based on imaging modality and segmentation methodology can be systematically evaluated at the bone piece level. A gradual transition towards the subsystem and system level will follow.

Given the novelty of the subject-specific approach, it is advisable to compare simulated full-system responses with experimental data early in the process to establish error benchmarks. Building on this foundation, subsequent work could employ more advanced modeling strategies, with particular emphasis on anatomically complex regions such as the base of the skull.

Regarding material characterization, a mathematically precise representation of the BC excitation is essential for accurate simulation-based parameter fitting at the bone piece and cortical layer levels. Potential errors introduced by experimental procedures, such as sample preparation and boundary condition variability, should be quantified to define the applicable validation range. In parallel, micromechanical characterization of the frequency-dependent properties of bone tissue could be performed to offer further insight into material behavior. Furthermore, as skin represents the second most influential material in BC transmission, its characterization should be prioritized in future studies.

To obtain statistically meaningful conclusions, validated methodologies should be applied across larger sample sizes. Considering the potential for future advances in data processing techniques and the inherently subject-specific nature of the data, the implementation of a relational database system would provide substantial long-term benefits for data management and reuse.

With access to a sufficiently large cadaveric head dataset, correlation of dynamic properties to (hydrated) meso- and microstructure of bone could lead to the identification of structural parameters governing macroscopic behavior. From this, principles for the design of metamaterials can be derived.

On the system level, model accuracy could reach a level of reliability suitable for in vivo patient-specific simulations. This would represent a substantial advance toward enabling personalized treatment strategies. However, experimental procedures would need to be adapted for in vivo applications, particularly with respect to imaging protocols (e.g., radiation dose) and validation metrics, owing to ethical and practical considerations. In the interim, the methodology that has already been developed could be used to replicate cadaveric head experiments through numerical simulations, thereby supporting both fundamental research and the continued development of BCHAs.

## Figures and Tables

**Table 1:** Overview of mass and time measurements for the three experimental conditions applied to the PMR sample.

Sample Condition	Bone Piece Wet	Bone Piece Dry	Cortical Layer Dry
Mass before measurement	29.36 g	22.81 g	8.11 g
Mass after measurement	28.96 g	22.83 g	8.12 g
Mass change (%)	−1.36	+0.09	+0.12
Measurement time	46 min	50 min	33 min

**Figure A1:**
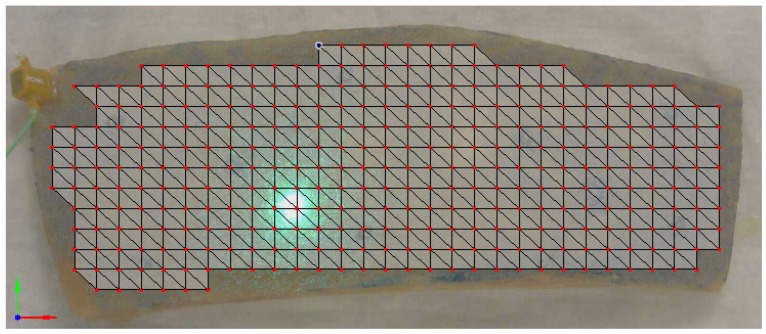
PMR wet bone piece with PA.

**Figure A2:**
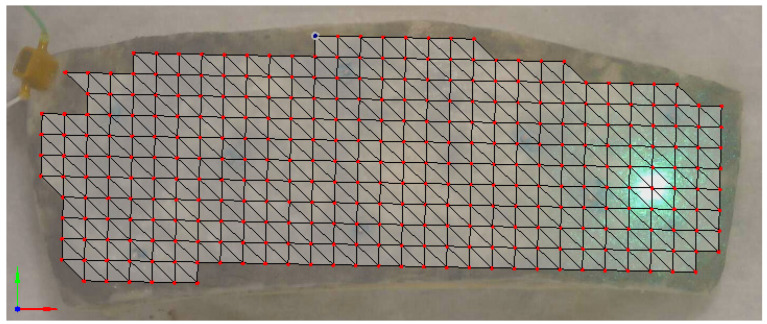
PMR dry bone piece with PA.

**Figure A3:**
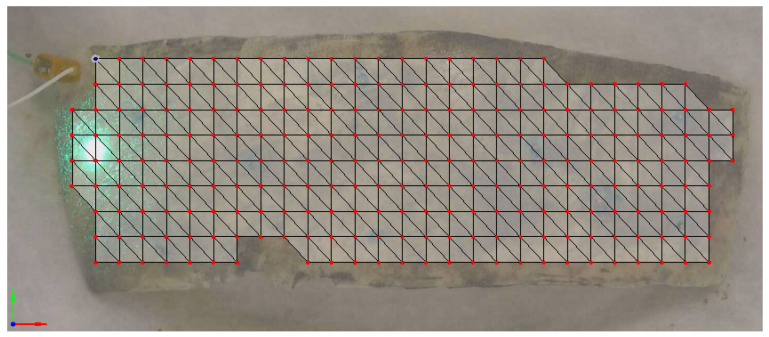
PMR dry cortical layer with PA.

## Data Availability

Dataset available on request from the authors.
